# Data Size and Quality Matter: Generating Physically-Realistic Distance Maps of Protein Tertiary Structures

**DOI:** 10.3390/biom12070908

**Published:** 2022-06-29

**Authors:** Fardina Fathmiul Alam, Amarda Shehu

**Affiliations:** 1 Department of Computer Science, George Mason University, Fairfax, VA 22030, USA; falam5@gmu.edu; 2 Center for Advancing Human-Machine Partnerships, George Mason University, Fairfax, VA 22030, USA; 3 Department of Bioengineering, George Mason University, Fairfax, VA 22030, USA; 4 School of Systems Biology, George Mason University, Fairfax, VA 22030, USA

**Keywords:** protein molecule, tertiary structure, multi-structure view, generative model, variational autoencoder, spatial pyramidal pooling, training set configuration, disentanglement

## Abstract

With the debut of AlphaFold2, we now can get a highly-accurate view of a reasonable equilibrium tertiary structure of a protein molecule. Yet, a single-structure view is insufficient and does not account for the high structural plasticity of protein molecules. Obtaining a multi-structure view of a protein molecule continues to be an outstanding challenge in computational structural biology. In tandem with methods formulated under the umbrella of stochastic optimization, we are now seeing rapid advances in the capabilities of methods based on deep learning. In recent work, we advance the capability of these models to learn from experimentally-available tertiary structures of protein molecules of varying lengths. In this work, we elucidate the important role of the composition of the training dataset on the neural network’s ability to learn key local and distal patterns in tertiary structures. To make such patterns visible to the network, we utilize a contact map-based representation of protein tertiary structure. We show interesting relationships between data size, quality, and composition on the ability of latent variable models to learn key patterns of tertiary structure. In addition, we present a disentangled latent variable model which improves upon the state-of-the-art variable autoencoder-based model in key, physically-realistic structural patterns. We believe this work opens up further avenues of research on deep learning-based models for computing multi-structure views of protein molecules.

## 1. Introduction

The key relationship between protein tertiary structure and protein function has motivated decades of computational research on predicting in silico an equilibrium tertiary structure of a protein molecule [1]. The community of researchers organized around such efforts through the CASP biennial competition [2] made seminal developments over the years, of which we highlight ResNet and AlphaFold2. Changing the focus from optimization-based approaches built over the molecular fragment replacement technique [3] to predicting contacts with deep neural networks was a key advancement [4]. In particular, ResNet [5] represented a significant advance in our ability to compute amino-acid contacts through deep neural networks leveraging sequence covariation and trained over known protein sequences. ResNet became a precursor to DeepMind’s AlphaFold2 [6], which is now hailed as a solution to a fifty-years-old grand challenge in computational biology.

With AlphaFold2 we now can get a highly-accurate view of a reasonable equilibrium tertiary structure of a protein molecule. Yet, we have known for decades that a single-structure view is insufficient to understand the relationship between structure and function, as it ignores the importance of structural dynamics as a key regulator of molecular interactions in the cell [7]. However, accounting for dynamics and obtaining a multi-structure view of a protein molecule continues to be an outstanding challenge in computational structural biology.

While a review of literature on computing a multi-structure view is beyond the scope of this paper, it is worth noting that this literature is rich in concepts and techniques developed and refined over decades of work on this fundamental problem. Methods formulated under the umbrella of Molecular Dynamics (that is, numerical simulation), Monte Carlo, or both via hybridization abound [8]. These methods have been able to provide highly-accurate and detailed views of the functionally-relevant structure space of a protein molecule, but overall they are either explicitly limited to a particular molecule or class of molecules or shown to be less effective in their capability to generalize [1]. The key insight for this shortcoming is that in order to handle the high dimensionality of the underlying search space, these methods often utilize key insight specific to a system of interest [9,10,11,12].

The effectiveness of deep learning-based methods for single-structure prediction [13,14,15,16] is further motivating researchers to seek related platforms for expanding the static view to the dynamic view. While work on this is largely beginning, various generative deep models can be found in literature [17]. They aim to learn directly from tertiary structures typically represented as contact maps or distance matrices through primarily variational autoencoders (VAEs) [18,19] or generative adversarial networks (GANs) [20,21,22]. Until recently [23], the majority of these models were limited to learning from same-length protein fragments. Work in [23] employs the technique of spatial pyramidal pooling in conjunction with convolutional layers to obtain a VAE, referred to as CVAE-SPP, capable of learning from experimental tertiary structures of protein molecules of any length obtained from the Protein Data Bank [24]. The rigorous evaluation demonstrates that CVAE-SPP is capable of capturing local and distal patterns of tertiary structures (namely, short- and long-range contacts) better than existing models.

When accommodating different-size objects in the training dataset, one has to be aware of the possible implications of the composition of the training dataset for the quality of generated data. In this paper we investigate this along various dimensions; utilizing sequence-non-redundant datasets of crystal tertiary structures, we investigate the impact of dataset size, quality, and composition on the ability of CVAE-SPP to reproduce physically-realistic local and distal structural patterns in generated data. We evaluate the model on various training set configurations with regards to composition over different-length protein molecules. Finally, we extend CVAE-SPP to disentangle the learned latent factor by adding a regularization term in the loss function. The strength of the regularization is controlled through the β hyperparameter, as in the classic βVAE model. The resulting βCVAE-SPP is shown to improve over CVAE-SPP on various metrics of performance, further advancing the state of the art.

We hope the work we present in this paper opens up more avenues of research on generative deep models for computing multi-structure views of protein molecules. Many future directions of research promise to sustain these avenues, including end-to-end models that do not limit the models to work with intermediate representations of tertiary structures but rather tertiary structures themselves.

## 2. Related Work

Many deep generative models for protein structure modeling can now be found in literature [18,22,25,26,27,28,29,30]. Some of the earliest work [27,28] demonstrated the ability of a vanilla convolutional GAN to learn over fixed-size distance matrices, with the latter capturing pairwise Euclidean distances of the central carbon atoms in amino acids. The analogy leveraged was between a distance matrix and a pixel image.

While rigorous metrics would be introduced later [22], the qualitative analysis demonstrated varying performance. One issue identified was the difficulty of GAN-based models to focus on all patterns present in distance matrices or contact maps (binarized versions of distance matrices based on a proximity threshold). Various works followed seeking to address this issue. Some relied on specializing the loss function so as to focus the network to learn the symmetry of contact maps [20]. Others utilized the loss function to focus on the sparse, long-range contacts [21].

Work in [22] evaluated GAN-based models and showed that the generated distance and contact matrices were often not physically-realistic for various reasons. Key metrics, utilized in this paper, as well, elucidated the varying performance on capturing the backbone, the short-range contacts, and the long-range contacts by different models. Work in [22] also showed that training GANs presented many challenges, and proposed various training mechanisms to stabilize the models, as well as a Wasserstein GAN to improve the quality of generated distance matrices.

In contrast to the dominant GAN architecture in literature, work in [31] explored the vanilla VAE architecture and showed promising results. Work in [23] extended this architecture with convolutional layers and the spatial pyramidal pooling technique to account for contact maps of different sizes and so leverage tertiary structures of proteins of varying amino-acid sequence lengths. The CVAE-SPP proposed in [23] provides the foundational architecture upon which we build in this work. As summarized in Section 1, we evaluate the impact of training dataset size, quality, and composition on the ability of this architecture to learn local and distal patterns in contact maps, as well as extend this architecture with disentanglement, resulting in a new model, βCVAE-SPP. Before proceeding with methodological details, we relate some preliminaries.

## 3. Preliminaries

### 3.1. 2D Representations of Tertiary Structure

The most popular 2D representations of a tertiary structure do away with the Cartesian coordinates of single atoms and instead record Euclidean pairwise distances. Typically, calculations are carried over the central carbon atom (CA) of each amino acid. In this manner, a tertiary structure of a protein molecule of *N* amino acids is summarized with an N×N distance matrix recording Euclidean distances of pairs of CA atoms. In a binarized version, a proximity threshold is introduced, which is typically 8 Å. Pairwise distances above this threshold are replaced with 0s, and the rest are replaced with 1’s. The result is referred to as a contact matrix, with 1-valued entries referred to as contacts. These 2D representations are translationally- and rotationally-invariant, so they are very popular for deep models. They also bring analogies with pixel images to the forefront, thus allowing one to leverage convolutional VAEs or GANs for generative tasks.

### 3.2. VAE Architecture

The SPP-CVAE architecture proposed in [23] is a foundational one that we evaluate along various dimensions and extend here, so we summarize it briefly for the reader. SPP-CVAE is a VAE at its core, so it consists of a parameterized encoder network, which is also referred to as the inference model, and a parameterized decoder network, which is referred to as the generative model. In general, in a VAE, each of these networks has one or more layers of units/neurons. The encoder maps the input layer *x* to the hidden/latent layer *y*. The decoder does the reverse and maps *y* to the output layer *z*, effectively reconstructing the data. The latent layer *y* is the latent representation that a VAE seeks to learn, and it contains the latent variables/factors.

The training of a VAE minimizes the difference between the reconstructed data (*z*) and input data (*x*). The key assumption in a VAE is that both the input and latent distributions are Gaussian. The encoder assumes that $x∼N(\mu_{x},\sigma_{x})$ exists. During training, the encoder learns the parameters $\mu_{x}$ and $\sigma_{x}$ of the input distribution. Similarly, VAEs assume that $z∼N(\mu_{x},\sigma_{x})$.

The VAE loss function combines a reconstruction term with a regularisation term; that is, $L={\left|x-y\right|}^{2}+KL\left(N\left(\mu_{x},\sigma_{x}\right)-N\left(0,I\right)\right)$. The first term is the reconstruction loss. The second term measures the Kullback-Leibler (KL) divergence between the returned distribution and a standard Gaussian distribution. More details on the VAE training process can be found in [32].

### 3.3. CVAE-SPP

**Figure 1:**
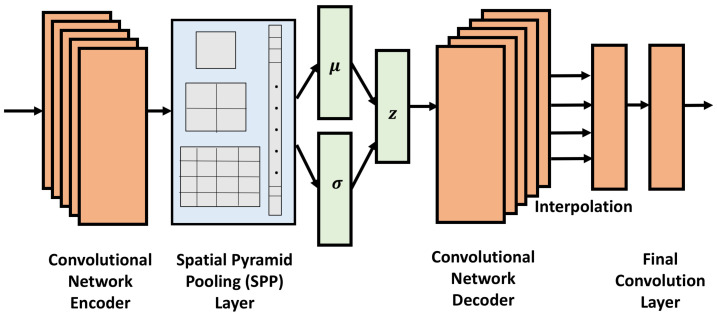
The key components of the proposed CVAE-SPP network are depicted in the schematic. Convolutional layers are used in both the encoder and decoder of the network, allowing it to learn patterns over distance matrices. A SPP layer enables the encoder to accommodate distance matrices of various sizes. Another proposed βCVAE-SPP network utilizes the same schematic but adds an extra hyperparameter denoted by β in order to regulate the loss function.

CVAE-SPP learns over distance matrices and so employs convolution layers in both the encoder and decoder. Figure 1 shows the architecture. Inspired by related work in computer vision [31,33], the architecture is extended with the SPP layer, so that the model can learn from distance matrices of varying sizes. As Figure 1 shows, the CVAE-SPP encoder uses a stack of convolutional layers followed by LeakyReLU for the activation function and Batch Normalization layers to support model optimization. An SPP layer follows the convolutional layer; in the SPP layer, each feature map is pooled multiple times. Its output vectors are concatenated into a single-dimensional vector that is then fed to a following fully-connected layer. The decoder consists of a stack of convolutional transpose layers coupled with LeakyReLU and Batch Normalization layers at the beginning. Each convolutional transpose layer increases the size of the height and width by a factor of two. At this point, the tensors generated are fixed in size and likely to be significantly smaller than the desired output. Interpolation addresses this issue by up-sampling or down-sampling the input to a desired size or scale. This layer aligns the corners of the data points and fills in the spaces by fitting various functions; we utilize the “nearest” mode, which enables data reshaping while allowing for backpropagation. A final convolutional layer is used to further improve the data reconstruction.

While more details can be found in [23], the SPP layer conducts pooling operations of varying sizes. Information “aggregation” is carried out at the beginning by splitting the input matrix into divisions from finer to coarser levels at a deeper stage, between the convolutional and fully-connected layers. The final convolutional layer’s feature maps are divided into multiple spatial bins, with the number of bins fixed. Each SPP layer has a resolution parameter *n*. Larger values indicate higher resolution and enable the pooling operation to capture smaller features. The sub-layers with larger values of *n* extract smaller features; the sub-layers with smaller values of *n* extract larger features. The effect is that of a pyramidal structure that makes the model input size-agnostic and enables it to learn from features of varying scales. The SPP layer in CVAE-SPP contains 3 sub-layers with corresponding *n* values of 1, 2, and 4.

## 4. Methods

### 4.1. β-CVAE-SPP

We first extend the CVAE-SPP loss function to force disentanglement on the latent factors and obtain a new model, β-CVAE-SPP. Following the β-VAE introduced in [34], β-CVAE-SPP augments the CVAE-SPP architecture with a regularization term, whose weight in the resulting loss function is controlled by the β hyperparameter. The loss function then becomes: $L={\left|x-y\right|}^{2}+\beta KL\left(N\left(\mu_{x},\sigma_{x}\right)-N\left(0,I\right)\right)$.

The hyperparameter β limits the latent bottleneck’s effective encoding capacity and encourages the latent representation to be increasingly factorised [35]. It is this factorization that we refer to as disentanglement.

The hyperparameter can be adjustable to strike a balance between the capacity of the latent channel and independence constraints with the precision of the reconstruction [34]. According to work in [34], the value of β should be greater than 1 so as to learn a disentangled latent representation. We investigate the impact of the value of β in a systematic manner. In a controlled experimental setting, we vary the value of this hyperparameter in a reasonable range and track the impact on performance along several metrics.

### 4.2. Training Dataset Compositions

Work in [23] shows that a model needs to see enough examples of a certain size before it is able to learn the inherent patterns in complex data, such as distance matrices (or contact maps). So, in this paper, we consider the following setting. First, from now on, we distinguish between an *input* dataset and a *training* dataset. A *training* dataset is what one can construct from an *input* dataset. For us, an *input* dataset will consist of tertiary structures obtained from the Protein Data Bank. However, rather than risk over- or under-representation of sequences and structures, we utilize the pre-curated datasets available by the PISCES server [36]. We related more details on these below. For now, let us refer to such a non-redundant input dataset of tertiary structures as *Input-Dataset*.

From this dataset, which invariably contains different-length proteins, one can choose to extract all fragments of *N* amino acids long and compute from these fragments the corresponding N×N distance matrices. One can vary *N* and so obtain different images of this dataset. We vary *N* in {64,72,90} in this paper in order to balance between sufficiently-long fragments and sufficiently-large training datasets. Specifically, we construct a training dataset where N=64. This is a dataset of fixed-size, 64×64 distance matrices, which refers as a baseline dataset. As one increases the value of *N*, the amount of data becomes scarcer. So, we consider varying compositions: a training dataset consisting of 64×64 and 72×72 distance matrices in a 70–30 split (so, 70% of the distance matrices are of size 64×64, and the rest are 72×72). In another training dataset, we vary this split to 50-50. In yet another, we introduce 90×90 distance matrices in a 40-30-30 split for N∈{64,72,90}. Finally, we consider the split 34–33–33. In summary, we consider 5 different training dataset configurations and Table 1 reports the descriptions. CVAE-SPP and βCVAE-SPP are trained on each of these five training datasets separately, and their generated distance matrices are evaluated for their realism along several metrics summarized below.

### 4.3. Input Datasets

We utilize separately three pre-compiled datasets, considering each as a representative, non-redundant view of the PDB obtained via the PISCES server [36]. What we control in the input datasets are tertiary structure quality; indirectly, the control on quality gives us control on the input dataset size, as well.

Specifically, we consider the following three input datasets, each containing tertiary structures of protein molecules varying in length from 40 to 1,440 amino acids: “cullpdb_pc15.0_res0.0-2.0_len40-10000_R0.25_Xray_d2022_02_22_chains3626”, “cullpdb_pc15.0_res0.0-2.5_len40-10000_Re0.3_Xray_d2022_02_22_chains4639”, and “cullpdb_pc15.0_res0.0-3.0_len40-10000_R0.3_Xray_d2022_02_22_chains5115”.

All three datasets have been extracted from the PDB on 22 February 2022. In each dataset, the percentage sequence identity cutoff is 15%, sequence lengths are between 40 and 10,000. The first dataset, which we abbreviate as “res0.0-2.0” from now on contains the best-quality tertiary structures. This control is executed through the upper bound of 2.0 in the X-ray resolution. Although R0.25 indicates a threshold on the quality of NMR structures, it is not utilized. The dataset contains only X-ray structures. This dataset contains the fewest structures, 3626 in all. In this dataset, the longest protein molecule contains 1440 amino acids.

The second dataset, which we abbreviate as “res0.0-2.5” worsens the resolution by increasing the upper bound; specifically, X-ray structures with resolution as low as 2.5 Å are allowed. Again, the dataset contains only X-ray structures, a total of 4639 structures. In this dataset, the longest protein molecule contains 1440 amino acids.

The third and last input dataset, to which we refer as “res0.0-3.0”, contains tertiary structures of even lower resolution (e.g., X-ray structures with resolution as low as 3.0 Å). This increases the dataset size further to 5116 structures. The dataset contains only X-ray structures. In this dataset, the longest protein molecule contains 1664 amino acids.

We note that for each of these datasets, the five different training configurations described above are utilized to generate five different training datasets. Training models on these datasets allows us to evaluate the impact of data size, quality, and composition on the quality of generated data.

### 4.4. Metrics to Evaluate Generated Data

Since generated data in our case are complex objectives, they can be summarized in different ways to obtain distributions that can then be compared with training data distributions to evaluate generated data quality. As in [22], we summarize each distance matrix with metrics that evaluate the presence of the backbone, short-range contacts, and long-range contacts.

*Backbone*: The presence of a backbone can be indicated by consecutive CA atoms at a distance of 3.83 Å from each-other, We expand this threshold to 4.0 Å as in [22] to allow for some variation from the ideal geometry that we also observed over experimental data. We first summarize a distance matrix with the percentage of consecutive CA atoms no further than this threshold; we refer to this summary metric as *% Backbone*.

*Short-range Contacts:* Here we count the number of [i,j] entries in the distance matrix (corresponding to Euclidean distances between the CA atom of amino acid at position *i* and the CA atom of amino acid at position *j*) whose values are no higher than 8 Å. We restrict to |j−i|≤4, which are referred to as short-range contacts. We refer to this metric as SR-Nr. Work in [22] introduces this metric for a fixed-size matrix. To account for distance matrices of varied sizes, we normalize this number by the number of CA atoms (dividing them by the number of rows of a distance matrix). We refer to this metric as *SR-Score*.

*Long-range Contacts:* Restring the above computations to |j−i|>4 provides us with the number of long-range contacts in a distance matrix. Normalizing by the number of CA atoms gives us the *LR-Score*.

Utilizing the above, each distance matrix can be then summarized with *% Backbone*, *SR-Score*, or *LR-Score*. We can do this for the training dataset and for the generated dataset, effectively obtaining distributions that then can be compared with any number of distance metrics. We utilize the Earthmover Distance (EMD) metric [37] here to compare a distribution over the training dataset to a distribution over the generated dataset. This comparison informs on whether a model has learned to reproduce in the generated data the local (Backbone, short-range contacts) and distal (long-range contacts) patterns observed over training data.

### 4.5. Statistical Significance Tests

In addition to comparing distributions over training and generated data and visualizing this comparison over training dataset configurations, we utilize statistical significance testing to make rigorous observations. We make use of the SciPy library [38]. The Supplementary Materials contains a large number of varying tests that we carry out, each with its own assumptions on the underlying distribution of the data and various models for multi-test correction and more. In Section 5 we only relate two representative tests. The first one is the One-way Analysis of Variance (ANOVA) which is a statistical technique used to determine whether the means of two or more samples are significantly different or not using the F distribution [39]. The ANOVA evaluates the null hypothesis, which assumes that all samples in all compared groups are derived from populations with identical mean values. To accomplish this, two estimates of the population variance are generated considering various assumptions. The ANOVA yields an F-statistic, the variation between means divided by the variance within samples. According to the central limit theorem, if the group means are chosen from the same population, their variance should be less than the sample variance. A larger ratio indicates that the samples came from populations with diverse mean values.

The second test we relate in Section 5 is a Kruskal-Wallis test [40] considered to be the non-parametric equivalent of the One-Way ANOVA test. A significant Kruskal–Wallis test implies that at least one sample stochastically dominates another sample. This can be shown by comparing the means of at least independent groups of two samples.

We conduct Post hoc tests after the statistical significance tests. Post hoc analysis, also known as a posteriori analysis, is a sort of statistical analysis that is performed after the rejection of the null hypothesis. The most common application of post hoc analysis is to investigate mean differences. When we run a statistical significance test to compare two or three group means, the findings may show that not all group means are equal but does not help identify which variations between means are significant. Using Post hoc tests, it is possible to compare several group means while adjusting the experiment-wise error rate. There are several techniques to conduct a post-hoc analysis (Holm, Bonferroni, Hommel, Hochberg, Nemenyi, etc.). We choose Dunn’s Test [41] with 2-stage FDR (false discovery rate) Benjamini-Hochberg and Holm-Bonferroni methods, as these are robust and established statistical procedures to justify the performance of the models. We note that Dunn’s Test is a multiple comparison test used to pinpoint which specific means are significant from the others. Both the Benjamini-Hochberg and Holm-Bonferroni methods are effective in lowering the FDR and avoiding Type I errors (false positives) [42]. In this work, we only show a subset of the post-hoc analysis results; the others are included in the Supplementary Materials.

### 4.6. Implementation Details

All models are implemented, trained, and evaluated using Pytorch Lightning [43] which is an open-source python library that provides a high-level interface for the PyTorch deep learning framework. We trained each of the investigated models for a total of 90 epochs; we observe that all models converge at around 40 to 45 epochs. For both of our VAE models, we use a batch size of 32 when all the distances matrices are of the same size (64×64). For the other four configurations of the training dataset, we consider a batch size of 1 to handle input data of variable-lengths and to avoid having different sizes in the same batch. To avoid premature convergence, a learning rate of 0.0003 is utilized. Training times for the models vary from 1500.143 to 2700.238 s. After a model is trained, we utilize its decode to generate distance matrices.

## 5. Results

We first evaluate the impact of varying β in βCVAE-SPP on the quality of generated data. We then compare βCVAE-SPP to CVAE-SPP in terms of the quality of the data they generate. In each case, the evaluation compares the generated to the training data via EMD along each of the three metrics related in Section 4. We additionally isolate and evaluate the impact of data quality, size, and composition on the quality of generated data. Finally, we relate the findings of statistical significance tests that compare models, input data, and dataset configurations on the quality of generated data.

### 5.1. Impact of β in βCVAE-SPP

**Figure 2:**
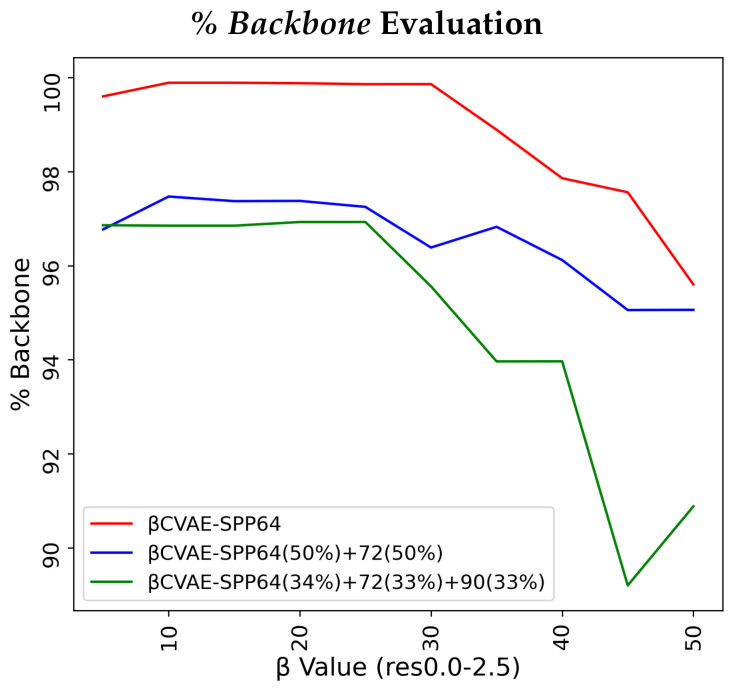

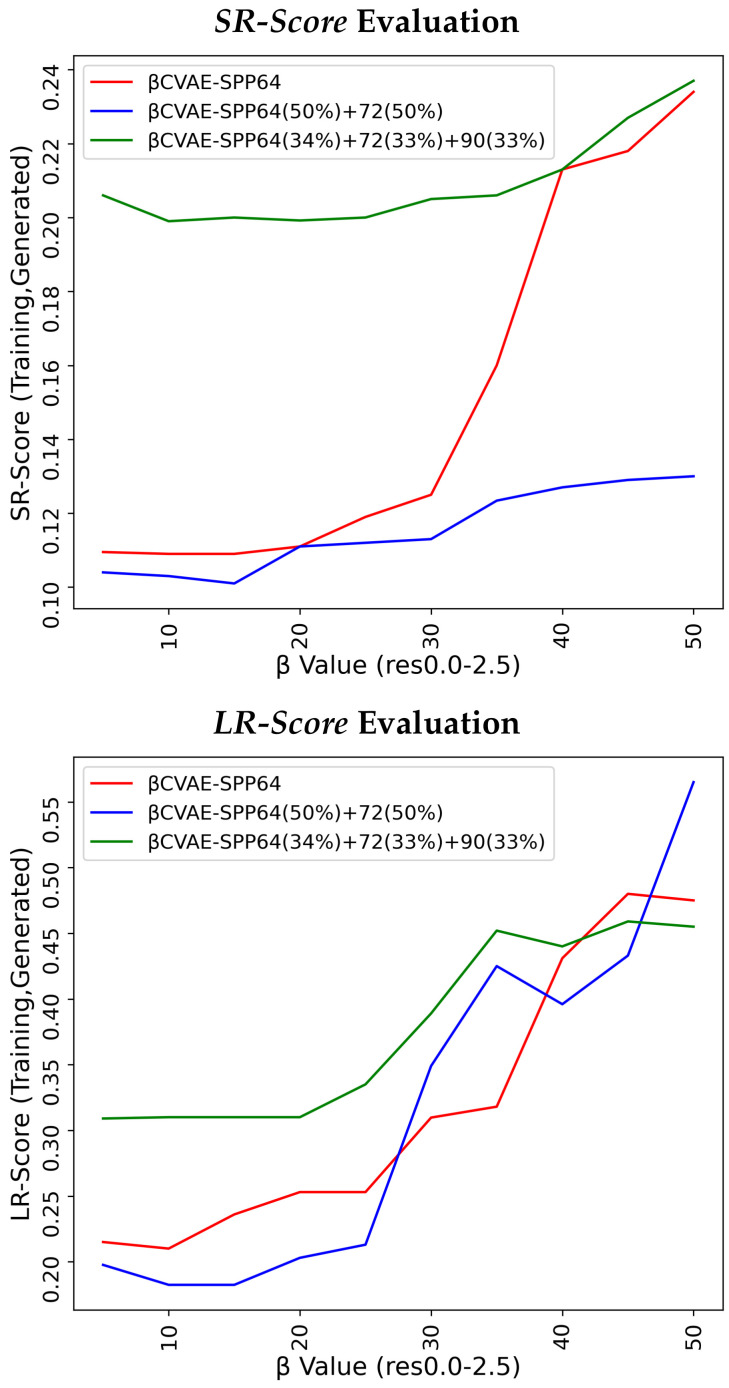
Varying β in [5,50] in increments of 5. Each resulting β-CVAE-SPP model is trained separately on each of the 3 training dataset configurations built over the res0.0-2.5 input dataset. Data generated by each model are compared to the respective training dataset via EMD over *% Backbone* (top panel), *SR-Score* (middle panel), and *LR-Score* (bottom panel).

In this experiment, we restrict our focus to the res0.0-2.5 input dataset. We vary β∈[5,50] in increments of 5. We train each resulting model on 3 of the 5 training dataset configurations separately, and then compare the data generated by each model to the respective training dataset via EMD along *% Backbone*, *SR-Score*, and *LR-Score*. Figure 2 relates our findings. Figure 2 relates several interesting observations. As the value of the β hyperparameter grows, the percentage of reconstructed backbone decreases, whereas the *SR-* and *LR-Scores* of generated distance matrices improve. The disentanglement term applies more pressure away from reconstruction in the loss function. Generally, all models are for most β values above 90% in their reconstruction of the backbone and all achieve similar low EMD values on the *SR-* and *LR-Score* distributions. Notably, the training dataset configuration that is more diverse helps the models most on recovering *SR-* and *LR-*patterns (green lines) for the majority of β values. Considering these results altogether, we fix the value of β to 10 for βCVAE-SPP in the rest of the experiments.

### 5.2. Comparison of βCVAE-SPP to CVAE-SPP

**Figure 3:**
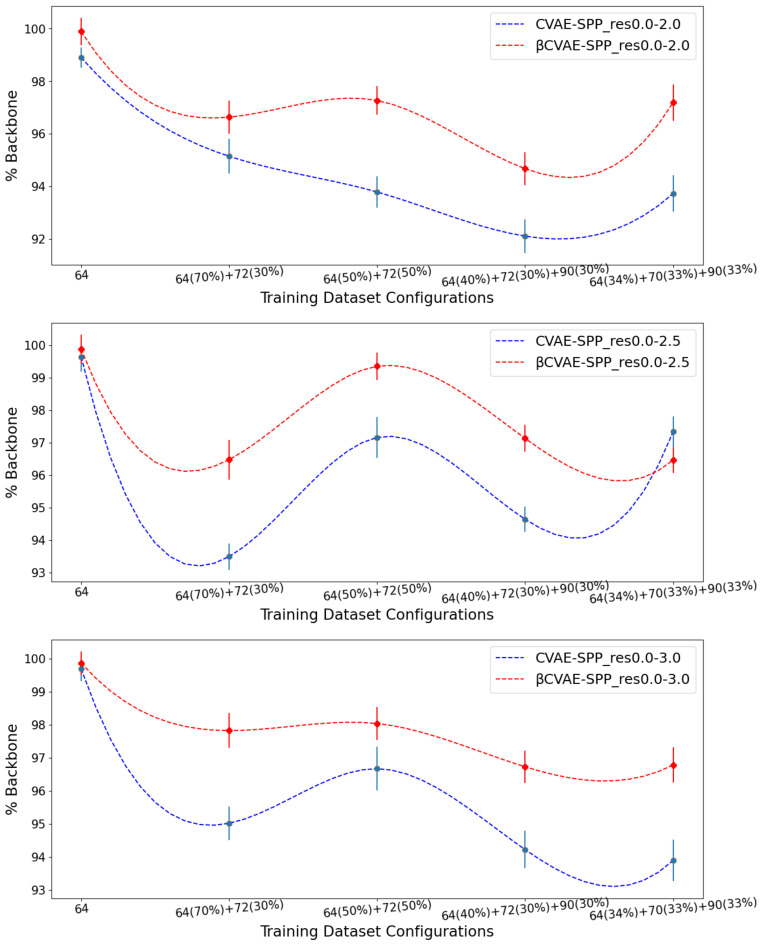
Average of *% Backbone* (over 3 independent runs) over generated distance matrices by CVAE-SPP and βCVAE-SPP. Each model is trained on a training dataset configuration (*x*-axis) of an input dataset. The three panels relate the different input datasets.

**Figure 4:**
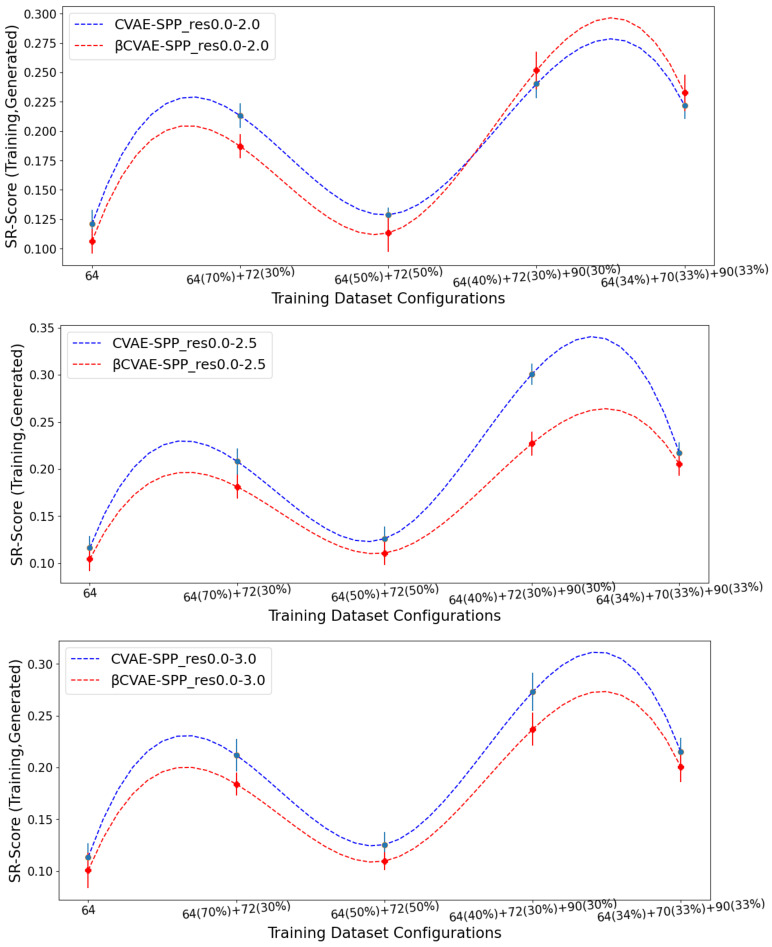
Average of EMD values over *SR-Score* distributions (over 3 independent runs) computed over generated versus training distance matrices by CVAE-SPP and βCVAE-SPP. Each model is trained on a training dataset configuration (*x* axis) of an input dataset. The three panels relate the different input datasets.

**Figure 5:**
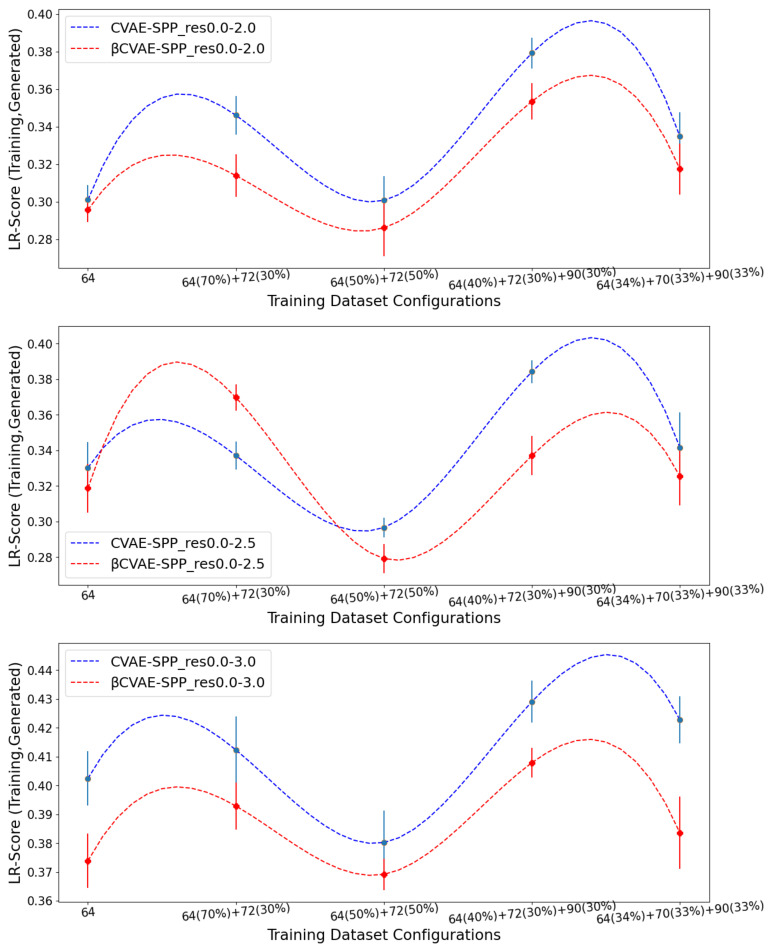
Average of EMD values over *LR-Score* distributions (over 3 independent runs) computed over generated versus training distance matrices by CVAE-SPP and βCVAE-SPP. Each model is trained on a training dataset configuration (*x* axis) of an input dataset. The three panels relate the different input datasets.

We now compare βCVAE-SPP to CVAE-SPP along each of the three structural metrics (*% Backbone*, *SR-Score*, and *LR-Score*). We do so on each of the three input datasets, on each of the five training dataset configurations per input dataset; note that this means we are effectively evaluating 15 trained models. To account for training variance, we train each model three times. We track the average performance of a model over the three runs but relate the standard deviation via error bars. Figure 3 relates the comparison over *% Backbone*. Figure 4 does so for the *SR-Score*, and Figure 5 does so for the *LR-Score*.

Figure 3 clearly shows that βCVAE-SPP outperforms CVAE-SPP for most of the training configurations. In particular, while both models start with a similar performance on the training dataset configuration containing only 64×64 distance matrices, the differentiation in performance becomes visible as the training dataset becomes more diverse. An exception is observed on the res0.0-2.5 input dataset, at the 34–33–33 training dataset configuration. We note that for all models and all settings, the *% Backbone* reconstruction is at a high value of 93%.

Figure 4 and Figure 5 suggest that βCVAE-SPP and CVAE-SPP are closer in performance in terms of the short-range and long-range contacts. In particular, Figure 4 shows that the differences between βCVAE-SPP and CVAE-SPP, which we note are EMD values, are small. Interestingly, irregardless of the model, the 64×64 and the 50–50 split are the two training dataset configurations that confer the best performance (lowest EMD values). This trend holds also for the evaluation over long-range contacts, related in Figure 5.

Altogether, these results suggest that there are few differences among the models, and that, interestingly, the addition of disentanglement does not hurt the model. In fact, it even improves its ability to recover more of the backbone in generated data.

### 5.3. Evaluating the Impact of Data Size, Quality, and Composition

We now present the comparisons related above in such a way as to expose, if present, any impact on generated data quality by the input datasets. Similar to the above analysis, we relate the average *Backbone* % over the generated dataset and then the EMD values comparing the generated to the training dataset over *SR-* and *LR-Score*.

**Figure 6:**
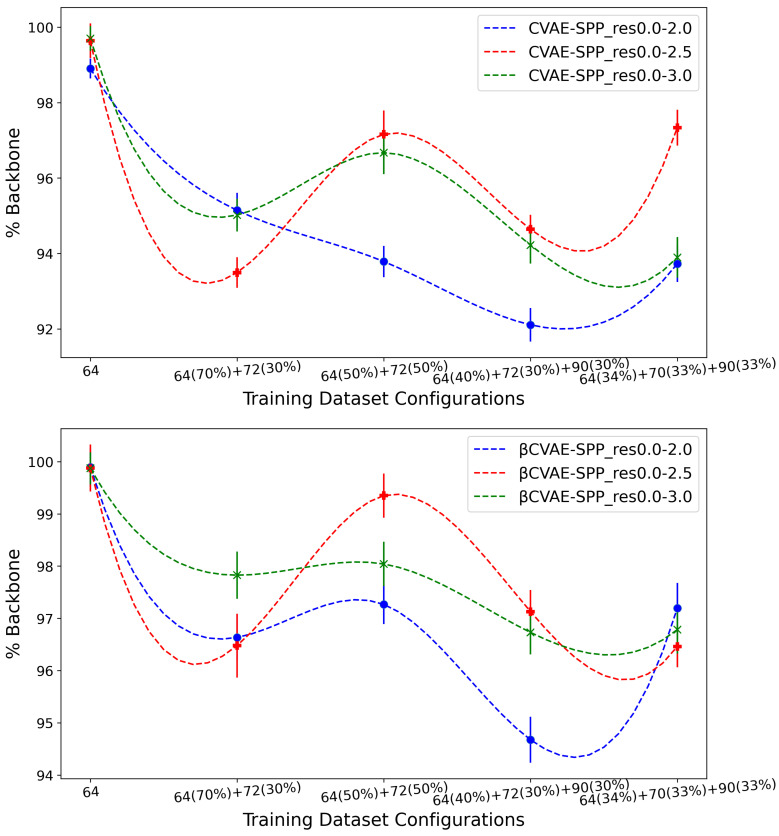
The (average) percentage of backbone over generated data for the CVAE-SPP (top panel) and βCVAE-SPP model (bottom panel) is compared along the five training dataset configurations for each of the three input datasets.

Figure 6 shows the (average) percentage of backbone over generated data for the CVAE-SPP (top panel) and βCVAE-SPP model (bottom panel) along the training dataset configurations for each of the three input datasets (denoted by curves of different colors; blue for res0.0-2.0, red for res0.0-2.5, and green for res0.0-3.0). Figure 6 shows a similar trend for each of the models; as the training dataset diversity increases, the average *% Backbone* goes down, with a small increase observed over the most diverse dataset. Additionally comparing the three input datasets reveals that the highest performance is obtained from the res0.0-2.5 input dataset and the 50–50 training dataset configuration. Interestingly, the differences due to the three input datasets largely disappear on the most diverse training dataset configuration when β-CVAE-SPP is utilized.

**Figure 7:**
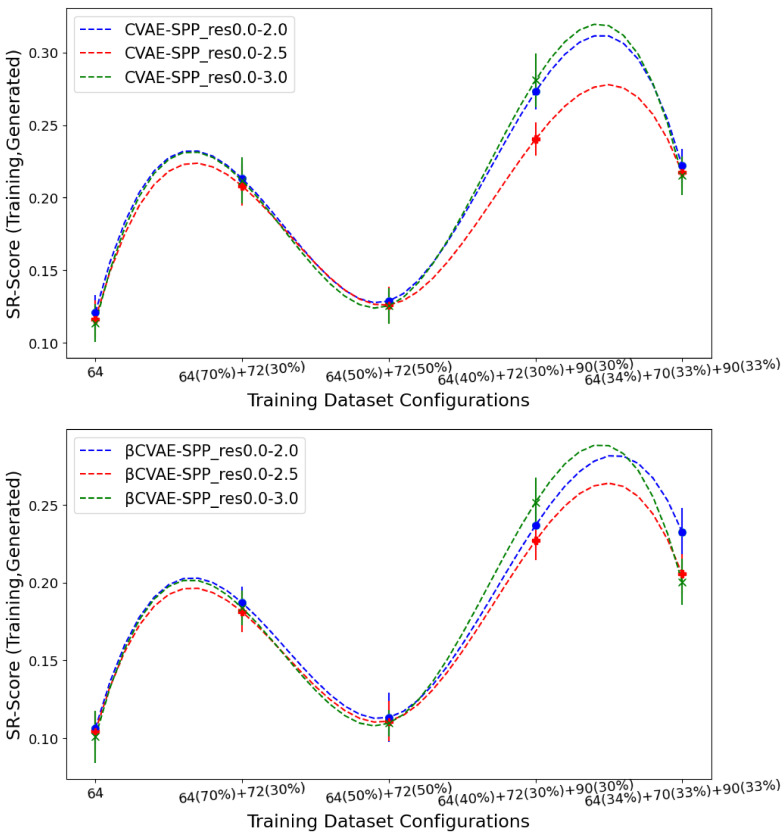
The EMD over *SR-Score* distributions of generated versus training data are tracked separately for CVAE-SPP (top panel) and βCVAE-SPP. In each panel, the comparison is made along the five training dataset configurations for each of the three input datasets.

Figure 7 tracks the EMD value over *SR-Score* distributions of generated versus training data separately for CVAE-SPP (top panel) and βCVAE-SPP. In each panel, the comparison is made along the five training dataset configurations for each of the three input datasets. Figure 7 shows that the input dataset largely has no bearing on the short-range contacts. Tracking along the training dataset configurations however, clearly shows that the 64×64 configuration confers the best performance, followed closely by the 50–50 split.

**Figure 8:**
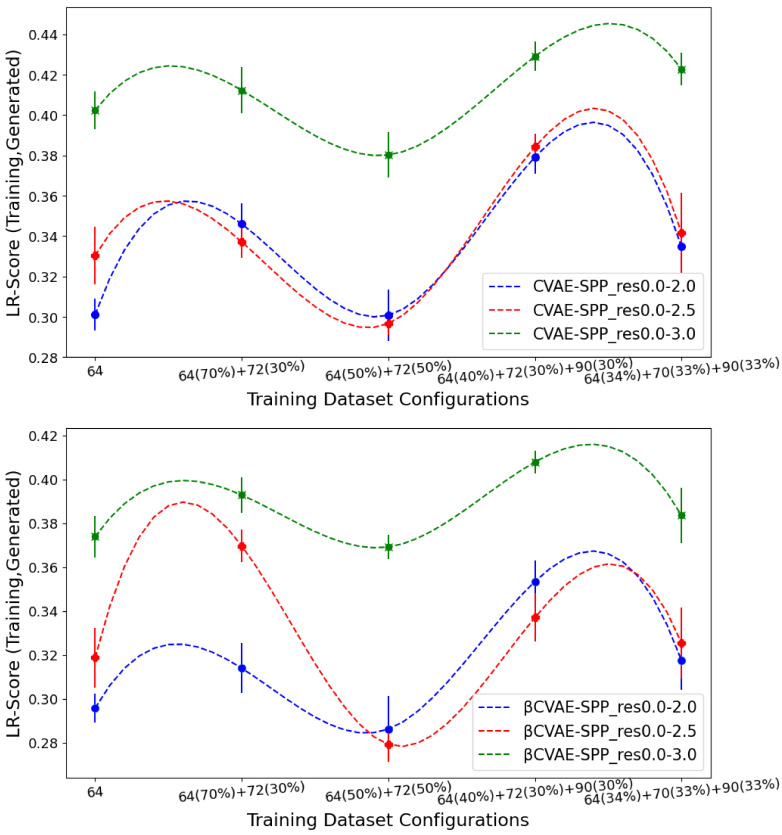
The EMD over *LR-Score* distributions of generated versus training data are tracked separately for CVAE-SPP (top panel) and βCVAE-SPP. In each panel, the comparison is made along the five training dataset configurations for each of the three input datasets.

Figure 8 now tracks the EMD value over *LR-Score* distributions of generated versus training data separately for CVAE-SPP (top panel) and βCVAE-SPP. In each panel, the comparison is made along the five training dataset configurations for each of the three input datasets. Unlike the results above, Figure 8 shows that the input dataset impacts the ability of the models to reconstruct long-range contacts as in the training dataset. In particular, the res0.0-3.0 dataset confers the worst performance (highest EMD values over all training dataset configurations for both models). We recall that the quality of the tertiary structures in this input dataset is worse, and this seems to impact the ability of both CVAE-SPP and βCVAE-SPP to learn realistic long-range contact distributions. Largely, the differences due to the other two datasets are smaller. As above, for short-range contacts, tracking along the training dataset configurations clearly shows that the 64×64 configuration confers the best performance (lowest EMD value), followed closely by the 50–50 split.

### 5.4. Visualization of Distance Matrices as Heatmaps

**Figure 9:**
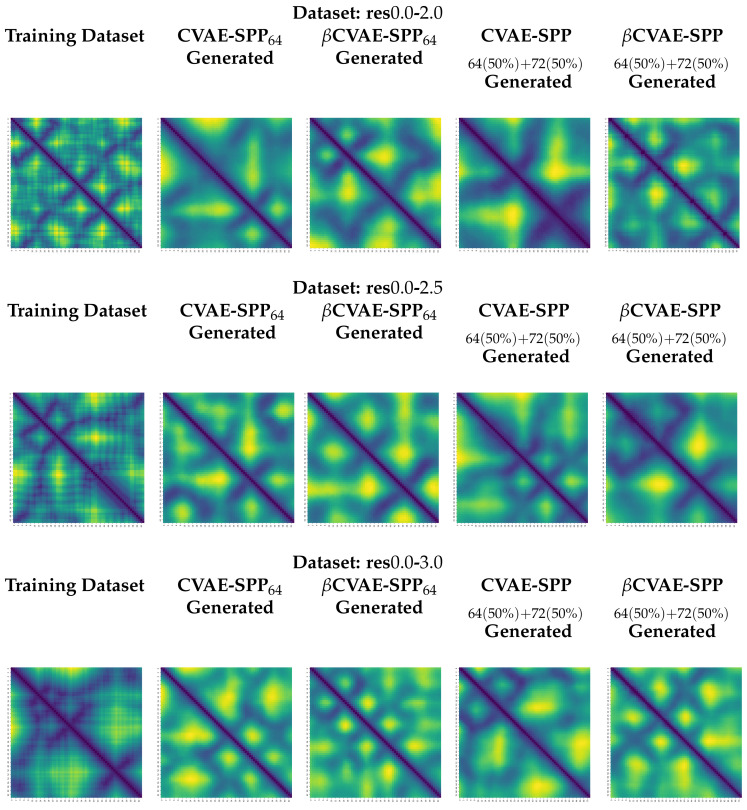
We relate here distance matrices selected at random over a training dataset (leftmost column) and over generated datasets (other columns) for each model and two of the training dataset configurations. The rows separate the three input datasets utilized. A yellow-to-blue color spectrum indicates high-to-low distance values.

We visualize generated distance matrices by the top two training dataset configuration settings indicated by the analysis above, 64×64 and 50–50 composition setting. We do so for each input dataset, and each model, in addition to the training dataset configuration. We select three distance matrices at random in each setting. These distance matrices are related as heatmaps in Figure 9. The heatmaps employ a yellow-to-blue color scheme to visually relate higher-to-lower distances. The existence of the backbone in the distance matrices as a dark line running along the main diagonal is clear. Short-range and long-range contacts as dark lines off the main diagonal are also visible. Visually, the generated distance matrices resemble actual distance matrices expected of tertiary structures.

### 5.5. Statistical Significance Analysis

Since the above analysis suggests possible differences over long-range contacts, we restrict the statistical significance analysis here to those contacts. As is typical, we consider α = 0.05.

Table 2 reports the results of the one-way ANOVA test and its non-parametric equivalent, the Kruskal–Wallis test. We conduct each test separately on CVAE-SPP and βCVAE-SPP. For each model, we compare 3 groups that allow us to evaluate the impact of the input dataset (res0.0-2.0 vs. res0.0-2.5 vs. res0.0-3.0). Each group contains the EMD values obtained over all five training dataset configurations; so each group contains 5 values. Each value is the EMD measuring the distance between the *LR-Score* distribution over the generated and the *LR-Score* distribution over the training dataset. Table 2 shows that all obtained *p*-values are under the α value; that is, the means are different and that this is statistically significant. This result holds by both the One-way ANOVA test and the Kruskal-Wallis test. This result confirms our visual observations above; that is, there are differences due to the three different input datasets.

The above analysis does not locate the differences among the means. To do so, we conduct a post-hoc analysis after the null hypothesis is rejected, which is related in Table 2. As related in Section 4, we apply the Dunn’s multiple comparison test with the Benjamini-Hochberg method and the Holm-Bonferroni method to investigate differences between group means. Table 3 relates all pairwise comparisons across the input datasets. The highlighted *p*-values indicate statistically-significant differences.

Table 3 shows that when CVAE-SPP is employed, the Benjamini-Hochberg method shows that there are statistically-significant differences between the res0.0-3.0 and the res0.0-2.5 input datasets (we abuse terminology here, as the comparison is between means of EMD values obtained by trained models) and between the res0.0-3.0 and the res0.0-2.0 input datasets. No statistically-significant differences are observed between the res0.0-2.0 and the res0.0-2.5 input datasets. When the Holm-Bonferroni method is employed, the only statistically significant difference is between the res0.0-3.0 and the res0.0-2.0 input datasets. These observations are replicated in their entirety over the results obtained by βCVAE-SPP. Taken altogether, they suggest that indeed the input dataset impacts the quality of the generated data with regards to the realism of long-range contacts; statistically-significant differences are observed when the resolution worsens from 2.0 Å to 3.0 Å. These results clearly relate that dataset quality has an impact over the quality of data generated by a model.

Then we focus on for a specific dataset, whether the differences between CVAE-SPP and βCVAE-SPP are statistically significant or not. For each dataset, we compare 2 sets or groups of input. The distribution of the average of *LR-Score* values on 5 different configurations on the training dataset using CVAE-SPP model is the first set of input for a given dataset, and the distribution of the average of *LR-Score* values on 5 different configurations on the training dataset using βCVAE-SPP is the second set of input for that dataset.

Table 4 shows for each of the datasets (in row), we compare the distribution of the average of *LR-Score* values on 5 different configurations on the training dataset between CVAE-SPP and βCVAE-SPP models using different statistical significance tests and found that those difference are not statistically significant.

## 6. Conclusions

This paper advances research on deep generative models for computing multi-structure views of protein molecules. Building over state-of-the-art VAE frameworks and, in particular, architectures capable of accommodating different-length protein molecules, such as SPP-CVAE, we investigate here the impact of dataset quality, size, and composition. We utilize rigorous evaluation along various metrics gaging the presence of local and distal patterns expected in realistic tertiary structures. We additionally present a disentangled SPP-CVAE model and show that the disentanglement does not impact the quality of generated data; in fact, there is some benefit in the ability of the model to recover more of the backbone. Careful comparison of the models shows no meaningful differences on metrics related to short-range and long-range contacts; this conclusion is supported by statistical significance analysis related in the Supplementary Materials. Evaluation along the various input datasets shows that the quality of the dataset has a clear impact on the quality of generated data, and these observations are supported by statistical significance analysis. In particular, the analysis suggests that high-quality tertiary structures are needed to improve a model’s ability to capture realistic long-range/distal patterns.

The work presented in this paper opens up many avenues of future research, including end-to-end models that do not limit the models to work with intermediate representations of tertiary structures but rather tertiary structures themselves. Another important direction concerns making these models sequence-specific; that is, conditioning generated data on a given amino-acid sequence. We also note that graph-based representations of tertiary structures may open further directions of promising research on graph-based GANs, graphVAEs, and graph neural network architectures.

## Figures and Tables

**Table 1:** Descriptions of different training dataset configurations.

Training Dataset Configurations	Descriptions
Configuration 1: 64×64	All contact maps extracted from an input dataset are of size 64×64.
Configuration 2: 64(70%)+72(30%)	70% of the contact maps extracted from an input dataset are of size 64×64, and the rest are of size 72×72.
Configuration 3: 64(50%)+72(50%)	50% of the contact maps extracted from an input dataset are of size 64×64, and the rest are of size 72×72.
Configuration 4: 64(40%)+72(30%)+90(30%)	40% of the contact maps extracted from an input dataset are of size 64×64, 30% of the contact maps extracted from an input dataset are of size 72×72 and the rest are of size 90×90.
Configuration 5: 64(34%)+72(33%)+90(33%)	34% of the contact maps extracted from an input dataset are of size 64×64, 33% of the contact maps extracted from an input dataset are of size 72×72 and the rest are of size 90×90.

**Table 2:** Statistical significance test over 3 groups of EMD values (over LR-Scores of generated vs. training dataset distributions) corresponding to the three different input datasets. Each group includes EMD values over *LR-Score* distributions (generated versus training) obtained from a model trained over each of the five training dataset configurations. The test is repeated separately for CVAE-SPP and βCVAE-SPP. The non-parametric version of the One-way ANOVA test, the Kruskal Wallis test is included in the last column. *p*-values are shown. Those no higher than 0.005 are highlighted in bold, indicating that there are statistically-significant differences among the means of the three groups.

res0.0-2.0 vs. res0.0-2.5 vs. res0.0-3.0		
***LR-Score***		
Model	One way ANOVA	Kruskal-Wallis
	*p* value	*p* value
CVAE-SPP: 5 training dataset configs	**0.0017**	**0.0131**
βCVAE-SPP: 5 training dataset configs	**0.0018**	**0.0103**

**Table 3:** Post-hoc analysis over EMD values (over *LR-Score* generated vs. training dataset distributions) obtained over the training dataset configurations, comparing all pairs of input datasets. The analysis is carried out separately for CVAE-SPP and βCVAE-SPP. The left panel relates Dunn’s test using the FDR 2 stage Benjamini-Hochberg method. The right panel indicates Dunn’s test using the Holm-Bonferroni method. *p*-values are shown. Those no higher than 0.005 are highlighted in bold, indicating statistically-significant differences among the means of the groups under comparison.

Post Hoc Dunn’s Test (CVAE-SPP: 5 Different Configs on Training Dataset), α = 0.05							
FDR 2 stage Benjamini-Hochberg Method				Holm-Bonferroni Method			
*LR-Score* (Training, Generated)							
Dataset	res0.0-2.0	res0.0-2.5	res0.0-3.0	Dataset	res0.0-2.0	res0.0-2.5	res0.0-3.0
	*p* value				*p* value		
res0.0-2.0	1.0000	0.2958	**0.0067**	res0.0-2.0	1.0000	1.0000	**0.0266**
res0.0-2.5	0.2958	1.0000	**0.0066**	res0.0-2.5	1.0000	1.0000	0.3998
res0.0-3.0	**0.0067**	**0.0066**	1.0000	res0.0-3.0	**0.0266**	0.3998	1.0000
**Post Hoc Dunn’s Test (βCVAE-SPP: 5 Different Configs on Training Dataset), α = 0.05**							
FDR 2 stage Benjamini-Hochberg Method				Holm-Bonferroni Method			
*LR-Score* (Training, Generated)							
Dataset	res0.0-2.0	res0.0-2.5	res0.0-3.0	Dataset	res0.0-2.0	res0.0-2.5	res0.0-3.0
	*p* value				*p* value		
res0.0-2.0	1.0000	0.1598	**0.0037**	res0.0-2.0	1.0000	1.0000	**0.0112**
res0.0-2.5	0.1598	1.0000	**0.0141**	res0.0-2.5	1.0000	1.0000	0.0851
res0.0-3.0	**0.0037**	**0.0141**	1.0000	res0.0-3.0	**0.0112**	0.0851	1.000

**Table 4:** Statistical significance between CVAE-SPP and βCVAE-SPP models for each 3 datasets res0.0-2.0 (first row), res0.0-2.5 (second row) and res0.0-3.0 (third row) are determined through different statistical significance test at α = 0.05. Column 1 lists the individual dataset for CVAE-SPP and βCVAE-SPP models comparison. Column 2 shows the “*p*-value” using the One-way ANOVA test, Column 3 using the Student’s *t*-test, Column 4 using the Kruskal–Wallis test and Column 5 using the Mann-Whitney U test. We recall that *LR-Score* measures the number of long-range contacts in a distance matrix (normalizing by the number of CA atoms). And for both VAE models, we have considered all 5 different configurations on the training dataset.

CVAE-SPP vs. βCVAE-SPP: 5 Different Configs on Training Dataset					
***LR-Score***					
Dataset	One way ANOVA	*t*-test	Kruskal	Mann Whitney	Reject-hs
	*p* value				
res0.0-2.0	0.3409	0.3409	0.3472	0.4033	False
res0.0-2.5	0.5707	0.5707	0.3472	0.4033	False
res0.0-3.0	0.0624	0.0624	0.0758	0.0946	False

## Data Availability

Input data are obtained from http://dunbrack.fccc.edu/pisces/ (accessed on 4 March 2022).

## Supplementary Materials

The following supporting information regarding large number of varying statistical significance tests that we carry out, each with its own assumptions on the underlying distribution of the data and various models for multi-test correction and more can be downloaded at: https://www.mdpi.com/article/10.3390/biom12070908/s1. Refs. [44,45] are cited in Supplementary Materials.

## Abbreviations

The following abbreviations are used in this manuscript:

ANOVA	One-way Analysis of Variance
βVAE	Beta Variational Autoencoder
CA atom	Central Carbon atom
C-terminus	Carboxyl terminus
CASP	Critical Assessment of Protein Structure Prediction
CVAE	Convolutional Variational Autoencoder
EMD	Earth Mover Distance
GAN	Generative Adversarial Network
KL divergence	Kullback-Leibler divergence
LR	Long-Range Contact
N-terminus	$NH_{2}$ (Amino) terminus
PDB	Protein Data Bank
PISCES	Protein Sequence Culling Server
SPP	Spatial Pyramid Pooling
SR	Short-Range Contact
ResNet	Residual Neural Network
2-stage FDR	Two-stage False Discovery Rate
VAE	Variational Autoencoder
