# Supplementary material for: Data Size and Quality Matter: Generating Physically-Realistic Distance Maps of Protein Tertiary Structures
Source: Biomolecules. 2022 Jun 29;12(7):908. doi: 10.3390/biom12070908 (PMC9313347; doi:10.3390/biom12070908)
Supplement: Supplementary file 1 [file biomolecules-12-00908-s001.zip › biomolecules-1761779-supplementary.pdf]

Supplementary Material

0.1 Summary of Statistical Significant Testing

We utilize various statistical significance tests to rigorously compare methods and experimental settings. In summary, statistical significance is a mathematical method of demonstrating the reliability of a statistic [1], given the null hypothesis. In order to make decisions based on the results of any running experiments, we may need to verify that a relationship actually exists between the independent variables being studied. And different types of statistical significant tests can help to identify this relationship.

A finding is considered statistically significant when it is extremely unlikely to have occurred given the null hypothesis. More specifically, we define the significance level of a study as α which is the probability that the study will reject the null hypothesis if the null hypothesis is true. And the p-value 'p' of a result is the likelihood of getting a result at least as extreme, given that the null hypothesis is true. When $p \leq \alpha$, the finding is considered statistically significant. For our statistical significance testing, we consider $\alpha = 0.05$ according to the definition standard [2]. The null hypothesis is rejected if the p-value of an observed variable is less than or equal to the significance level. If the p-value is greater than the significance level, we cannot conclude that a difference is statistically significant.

0.2 Statistical Significance Tests Conducted in this Paper

Table S1 and S2 reports the results of the one-way Analysis of Variance (ANOVA) test and its non-parametric equivalent; the Kruskal–Wallis test, and Student's t-test. In Table S1, we conduct each test on βCVAE-SPP. For each model, we compare 2 groups that allow us to evaluate the impact of the input dataset (res0.0-2.0 versus res0.0-3.0). Each group contains the EMD values obtained over all five training dataset configurations; so each group contains 5 values. Each value is the EMD, measuring the distance between the *LR-Score* distribution over the generated and the *LR-Score* distribution over the training dataset. Table S2 follows the same procedure but instead of comparing two extreme dataset, we compare 3 groups that allow us to evaluate the impact of the input dataset (res0.0-2.0 versus res0.0-2.5 versus res0.0-3.0). And we conduct each test separately on CVAE-SPP and βCVAE-SPP. In both table, we want to determine whether there are any statistically significant differences between the means of these two or more independent (unrelated) groups, where our null hypothesis assumes that all means are equals.

Table S1 shows that when we compare βCVAE-SPP to itself by considering less extreme (res0.0-2.0) and highest extreme (res0.0-3.0) dataset in terms of noise and do this on average of distribution of *LR-Score* values obtained by 5 training dataset configurations using one-way analysis of variance (ANOVA) and Student's t-test, we found that in both cases, $p \leq \alpha$ (reject the null hypothesis) conclude that differences are statistically different, so we can say that disentanglement helps when quality of the dataset decreases.

We present more results in Table S2 in such a way as to expose, if present, any impact on generated data quality by the input datasets. Similar to the above analysis, we relate the EMD values comparing the generated to the training dataset over *LR-Score*. In order to analyze the impact of the input dataset, we

compare 3 groups for each model (res0.0-2.0 versus res0.0-2.5 versus res0.0-3.0). It contains all 5 training dataset configurations' EMD values, which means each group comprises 5 values. Table S2 shows that all obtained p-values are under the α value; which implies that the means are different and that this is statistically significant. This result holds by both the one-way ANOVA test and the Kruskal-Wallis test. This result confirms our visual observations in the main paper; that is, there are differences due to the three different input datasets.

Table S1: Statistical significance test over 2 groups of EMD values (over LR-Scores of generated versus training dataset distributions) corresponding to the 2 different input datasets (res0.0-2.0 and res0.0-3.0). Each group includes EMD values over *LR-Score* distributions (generated versus training) obtained from a model trained over each of the five training dataset configurations. The Student's t-test is included in the last column. P-values are shown. Those no higher than 0.005 are highlighted in bold, indicating that there are statistically-significant differences among the means of the three groups.

res0.0-2.0 vs res0.0-3.0		
LR-Score		
Model	One way ANOVA	T-test
	P value	P value
βCVAE-SPP 5 training dataset configs	**0.0006**	**0.0006**

Table S2: Statistical significance test over 3 groups of EMD values (over LR-Scores of generated versus training dataset distributions) corresponding to the three different input datasets. Each group includes EMD values over *LR-Score* distributions (generated versus training) obtained from a model trained over each of the five training dataset configurations. The test is repeated separately for CVAE-SPP and βCVAE-SPP. The non-parametric version of the one-way ANOVA test, the Kruskal Wallis test is included in the last column. P-values are shown. Those no higher than 0.005 are highlighted in bold, indicating that there are statistically-significant differences among the means of the three groups.

res0.0-2.0 vs res0.0-2.5 vs res0.0-3.0		
LR-Score		
Model	One way ANOVA	Kruskal-Wallis
	P value	P value
CVAE-SPP : 5 training dataset configs	**0.0017**	**0.0131**
βCVAE-SPP : 5 training dataset configs	**0.0018**	**0.0103**

The above analysis does not locate the differences among the means. To do so, we conduct several post-hoc analyses after the null hypothesis is rejected, which is related in Table S2. So, while controlling the experiment-wise error rate, we apply post hoc tests to investigate differences between different group averages.

We apply the Dunn's multiple comparison test with the Benjamini-Hochberg method and the Holm-Bonferroni method to investigate differences between group means in Table S3. Table S4 shows the results of applying post-hoc Tukey

HSD test and Table S5 for T-test Pairwise Comparison. All the tables relate all pairwise comparisons across the input datasets and the highlighted p-values indicate statistically-significant differences.

Table S3: Post-hoc analysis over EMD values (over *LR-Score* generated versus training dataset distributions) obtained over the training dataset configurations, comparing all pairs of input datasets. The analysis is carried out separately for CVAE-SPP and βCVAE-SPP. The panel relates Dunn's test using the FDR 2 stage Benjamini-Hochberg method. The right panel indicates Dunn's test using the Holm-Bonferroni method. P-values are shown. Those no higher than 0.005 are highlighted in bold, indicating statistically-significant differences among the means of the groups under comparison.

Post Hoc Dunn's Test (CVAE-SPP : 5 different configs on training dataset), α=0.05							
FDR 2 stage Benjamini-Hochberg Method				Holm-Bonferroni Method			
LR-Score (Training, Generated)							
Dataset	res0.0-2.0	res0.0-2.5	res0.0-3.0	Dataset	res0.0-2.0	res0.0-2.5	res0.0-3.0
	P value				P value		
res0.0-2.0	1.0000	0.2958	**0.0067**	res0.0-2.0	1.0000	1.0000	**0.0266**
res0.0-2.5	0.2958	1.0000	**0.0066**	res0.0-2.5	1.0000	1.0000	0.3998
res0.0-3.0	**0.0067**	**0.0066**	1.0000	res0.0-3.0	**0.0266**	0.3998	1.0000
Post Hoc Dunn's Test (βCVAE-SPP : 5 different configs on training dataset), α=0.05							
FDR 2 stage Benjamini-Hochberg Method				Holm-Bonferroni Method			
LR-Score (Training, Generated)							
Dataset	res0.0-2.0	res0.0-2.5	res0.0-3.0	Dataset	res0.0-2.0	res0.0-2.5	res0.0-3.0
	P value				P value		
res0.0-2.0	1.0000	0.1598	**0.0037**	res0.0-2.0	1.0000	1.0000	**0.0112**
res0.0-2.5	0.1598	1.0000	**0.0141**	res0.0-2.5	1.0000	1.0000	0.0851
res0.0-3.0	**0.0037**	**0.0141**	1.0000	res0.0-3.0	**0.0112**	0.0851	1.000

Table S3 shows that when CVAE-SPP is employed, the Benjamini-Hochberg method shows that there are statistically-significant differences between the res0.0-3.0 and the res 0.0-2.5 input datasets (we abuse terminology here, as the comparison is between means of EMD values obtained by trained models) and between the res0.0-3.0 and the res 0.0-2.0 input datasets. No statistically-significant differences are observed between the res0.0-2.0 and the res 0.0-2.5 input datasets. When the Holm-Bonferroni method is employed, the only statistically significant difference is between the res0.0-3.0 and the res 0.0-2.0 input datasets. These observations are replicated in their entirety over the results obtained by βCVAE-SPP.

The results in Table S4 using Tukey HSD post hoc analysis and in Table S5 using T-test Pairwise Comparison support the same conclusion as in Table S3 using Benjamini-Hochberg method show that there are statistically-significant differences between the res0.0-3.0 and the res 0.0-2.5 input datasets . Taken altogether, they suggest that indeed the input dataset impacts the quality of the generated data with regards to the realism of long-range contacts; statistically-significant differences are observed when the resolution worsens from 2.0Å to 3.0Å. These results clearly relate that dataset quality has an impact over the quality of data generated by a model.

Then we focus on for a specific dataset, whether the differences between CVAE-SPP and βCVAE-SPP are statistically significant or not. For each dataset,

Table S4: Post-hoc analysis using Tukey HSD test over EMD values (over *LR-Score* generated versus training dataset distributions) obtained over the training dataset configurations, comparing all pairs of input datasets. The analysis is carried out separately for CVAE-SPP (top panel) and βCVAE-SPP (bottom panel). Column 1 and 2 indicate datasets, and Column 3 shows compares means between group 1 and 2. In Colum 4, P-values are shown. Those no higher than 0.005 are highlighted in bold, indicating statistically-significant differences among the means of the groups under comparison.Columns 5 and 6 shows the lower and upper mean values. Columns 7 indicates whether the null hypothesis can be rejected or not. Boldface font indicates rejection of the null hypothesis.

Post Hoc Tukey HSD, FWER=0.05 Test (CVAE-SPP 5 training dataset configs)						
LR-Score (Training, Generated)						
Dataset (Group 1)	Dataset (Group 2)	Mean Diff.	P-Value	Lower	Upper	Reject
res0.0-2.0	res0.0-2.5	0.0056	0.9	-0.0425	0.0537	False
res0.0-2.0	res0.0-3.0	0.077	**0.0029**	0.0289	0.1252	**True**
res0.0-2.5	res0.0-3.0	0.0714	**0.005**	0.0233	0.1195	**True**

Post Hoc Tukey HSD, FWER=0.05 Test (βCVAE-SPP 5 training dataset configs)						
LR-Score (Training, Generated)						
Dataset (Group 1)	Dataset (Group 2)	Mean Diff.	P-adjust	Lower	Upper	Reject
res0.0-2.0	res0.0-2.5	0.0127	0.712	-0.0306	0.056	False
res0.0-2.0	res0.0-3.0	0.0722	**0.0021**	0.0288	0.1155	**True**
res0.0-2.5	res0.0-3.0	0.0595	**0.0084**	0.0162	0.1028	**True**

Table S5: Post-hoc analysis using T-test Pairwise Comparison test for critical values of mean differences over EMD values (over *LR-Score* generated versus training dataset distributions) obtained over the training dataset configurations, comparing all pairs of input datasets. The analysis is carried out separately for CVAE-SPP (top panel) and βCVAE-SPP (bottom panel). Columns 1 and 2 indicate datasets as group 1 and 2. Column 3 shows the coefficient, and Column 4 shows the standard error between groups 1 and 2. In Column 5, P-values are shown. Those no higher than 0.005 are highlighted in bold, indicating statistically-significant differences among the means of the groups under comparison. Column 6 indicates whether the null hypothesis should be rejected or not; Boldface font indicates rejection.

T-test Pairwise Comparison (CVAE-SPP 5 training dataset configs), α=0.05					
LR-Score (Training, Generated)					
Dataset (Group 1)	Dataset (Group 2)	Coef.	Std Err.	Pvalue-hs	Reject-hs
res0.0-2.0	res0.0-2.5	0.0056	0.0180	0.7598	False
res0.0-2.0	res0.0-3.0	0.0770	0.0180	**0.0032**	**True**
res0.0-2.5	res0.0-3.0	0.0714	0.0180	**0.0037**	**True**

T-test Pairwise Comparison (βCVAE-SPP 5 training dataset configs), α=0.05					
LR-Score (Training, Generated)					
Dataset (Group 1)	Dataset (Group 2)	Coef.	Std Err.	Pvalue-hs	Reject-hs
res0.0-2.0	res0.0-2.5	0.0126	0.0162	0.4503	False
res0.0-2.0	res0.0-3.0	0.0721	0.0162	**0.0024**	**True**
res0.0-2.5	res0.0-3.0	0.0594	0.0162	**0.0065**	**True**

Table S6: Statistical significance between CVAE-SPP and βCVAE-SPP models for each 3 datasets res0.0-2.0 (first row), res0.0-2.5 (second row) and res0.0-3.0 (third row) are determined through different statistical significance test at α =0.05. Column 1 lists the individual dataset for CVAE-SPP and βCVAE-SPP models comparison. Column 2 shows the "P-value" using the one-way ANOVA test, Column 3 using the Student's t-test, Column 4 using the Kruskal–Wallis test and Column 5 using the Mann-Whitney U test. We recall that *LR-Score* measures the number of long-range contacts in a distance matrix (normalizing by the number of CA atoms). And for both VAE models, we have considered all 5 different configurations on the training dataset.

CVAE-SPP vs βCVAE-SPP : 5 different configs on training dataset					
LR-Score					
Dataset	One way ANOVA	T-test	Kruskal	Mann Whitney	Reject-hs
	P value				
res0.0-2.0	0.3409	0.3409	0.3472	0.4033	False
res0.0-2.5	0.5707	0.5707	0.3472	0.4033	False
res0.0-3.0	0.0624	0.0624	0.0758	0.0946	False

we compare 2 sets or groups of input. The distribution of the average of *LR-Score* values on 5 different configurations on the training dataset using CVAE-SPP model is the first set of input for a given dataset, and the distribution of the average of *LR-Score* values on 5 different configurations on the training dataset using βCVAE-SPP is the second set of input for that dataset. Table S6 shows for each of the datasets (in row), we compare the distribution of the average of *LR-Score* values on 5 different configurations on the training dataset between CVAE-SPP and βCVAE-SPP models using different statistical significance tests and found that those difference are not statistically significant.

So we can conclude that differences across various datasets per model are statistically significant, whereas differences between models on any individual dataset are not statistically significant.

References

[1] Sirkin, R.M. *Statistics for the social sciences*; Sage, 2006.

[2] Sproull, N.L. *Handbook of research methods: A guide for practitioners and students in the social sciences*; Scarecrow press, 2002.
